# LST1: a novel biomarker for efferocytosis in the co-occurrence of type 2 diabetes mellitus and clear cell renal cell carcinoma

**DOI:** 10.3389/fimmu.2025.1737749

**Published:** 2025-12-19

**Authors:** Yuru Yang, Tingting Chen, Zhicheng Xu, Jialing Cai, Wenli Wang, Lan Pan, Xiaotian Cheng, Andong Wang

**Affiliations:** 1 School of Pharmacy, Nantong University, Nantong, Jiangsu, China; 2 Department of Pharmacy, Yancheng Clinical College of Xuzhou Medical University & First People’s Hospital of Yancheng, Yancheng, Jiangsu, China; 3 College of Traditional Chinese Medicine, Xinjiang Medical University, Urumqi, China

**Keywords:** biomarkers, clear cell renal cell carcinoma, machine learning, single-cell RNA sequencing, type 2 diabetes mellitus

## Abstract

**Background:**

The rising global incidence of clear cell renal cell carcinoma (ccRCC) often coincides with metabolic disorders like type 2 diabetes mellitus (T2DM). However, the association between these two conditions remains unclear. This study aimed to identify common molecular pathways and novel biomarkers for the comorbidity of T2DM and ccRCC.

**Methods:**

Utilizing single-cell transcriptomic datasets from the GEO public database, this study identified heterogeneous cell characteristics and core subpopulations in T2DM and ccRCC. Immune infiltration was assessed using the GSEA algorithm, and a prognostic model was optimized through machine learning algorithms. Key genes were pinpointed in conjunction with the optimal model’s score. Subsequently, *in vivo* and *in vitro* comorbidity models were established to validate the pivotal role of these key genes in the comorbidity.

**Results:**

Single-cell sequencing analysis revealed that efferocytosis was notably active in dead cell clearance in T2DM. In ccRCC, macrophages regulated efferocytosis to facilitate antigen presentation, modulate inflammation, and promote intercellular communication. Integrating machine learning and transcriptome analysis, we identified LST1 as a pivotal regulatory gene in both T2DM and ccRCC (AUC> 0.745). This result suggests that LST1 is involved in regulating macrophage-mediated efferocytosis and immune communication. Analysis of immune infiltration suggests that LST1-mediated efferocytosis may influence ccRCC susceptibility or disease progression by sustaining immune signaling activation and disrupting regulatory balance, potentially stemming from early inflammation in T2DM. Validation through *in vitro* and *in vivo* experimental models further underscores the critical role of LST1 in disease advancement.

**Conclusion:**

This study initially identified LST1 as a pivotal regulatory factor in the co-occurrence of T2DM and ccRCC, emphasizing its essential involvement in the immune interaction within the hyperglycemia-induced microenvironment of ccRCC. These results not only elucidated the immunomodulatory role of LST1 in individual diseases but also delineated an immunological continuum bridging diverse conditions, providing a novel framework for investigating the immune pathways implicated in the concurrent presence of diabetes and malignancies.

## Introduction

Renal cell carcinoma (RCC) is a prevalent malignant tumor arising from renal tubular epithelial cells, with clear cell renal cell carcinoma (ccRCC) being the most common subtype, accounting for over 70% of cases (1). Traditional treatment modalities encompass surgical resection and chemotherapy; however, the majority of patients are diagnosed at advanced stages (III or IV) when metastasis has occurred, significantly compromising cure rates and five-year survival rates (2). Additionally, a notable proportion of cancer patients present with concurrent metabolic disorders, with approximately 12% having diabetes (3). Patients with ccRCC and diabetes exhibit lower overall survival rates compared to non-diabetic counterparts, possibly due to the hyperglycemic microenvironment fostering ccRCC development and progression (4). Notably, the interplay between tumor microenvironment alterations and immune responses may underlie this association (5). In both ccRCC and T2DM, factors including inflammatory responses, angiogenesis, and immune evasion play pivotal roles in disease pathogenesis (6–8). Therefore, given the high mortality of ccRCC and the high prevalence of T2DM, recent studies have increasingly focused on whether T2DM exacerbates ccRCC progression (9). Hence, further exploration of potential biomarkers and elucidation of shared mechanisms and therapeutic targets between ccRCC and diabetes are imperative to advance our understanding of this comorbidity.

Single-cell RNA sequencing (scRNA-seq) is a rapidly advancing technology that enables the precise characterization of gene expression profiles in tens of thousands of individual cells, facilitating the classification of homogeneous cells into distinct subtypes or the discovery of novel cell types (10). Widely utilized across various disciplines including microbiology, immunology, neurology, and oncology, this technology shows promise in enhancing disease diagnosis, treatment, and prognosis (11). In ccRCC, scRNA-seq revealed notable alterations in the gene expression profiles of infiltrating immune cells, particularly an augmentation in CD8^+^ T cells and macrophages within the tumor microenvironment. Moreover, distinct transcriptional states of tumor-infiltrating CD8^+^ T cells were delineated, with the MKI67^+^ proliferative subset identified as a potential contributor to ccRCC pathogenesis (12). In the context of T2DM, scRNA-seq analysis identified three predominant cell clusters (neurons, epithelial cells, and smooth muscle cells) implicated in diabetes pathophysiology, particularly in insulin secretion and pancreatic development (13). Notably, the application of single-cell RNA sequencing (scRNA-seq) in investigating the comorbidity of T2DM and ccRCC comorbidity remains unexplored. This study leveraged scRNA-seq technology to comprehensively investigate two disease models, unveiling that early-stage T2DM, characterized by upregulated LST1 expression, triggers the activation of granulocyte efferocytosis and antigen presentation functions, perturbing immune homeostasis. This sustained microenvironmental shift enhances macrophage phagocytic, antigen presentation, and immune regulatory capacities during ccRCC progression, notably through HLA signal activation, ultimately fostering immune evasion and reshaping the tumor microenvironment. We therefore hypothesized that LST1-mediated efferocytosis forms an immunological bridge, connecting the chronic inflammatory milieu of T2DM to the immunosuppressive tumor microenvironment of ccRCC. To test this hypothesis, we designed a comprehensive study combining single-cell transcriptome analysis, machine learning, and *in vitro*/*in vivo* validation.

## Materials and methods

### Dataset collection of T2DM and ccRCC

**Figure 1:**
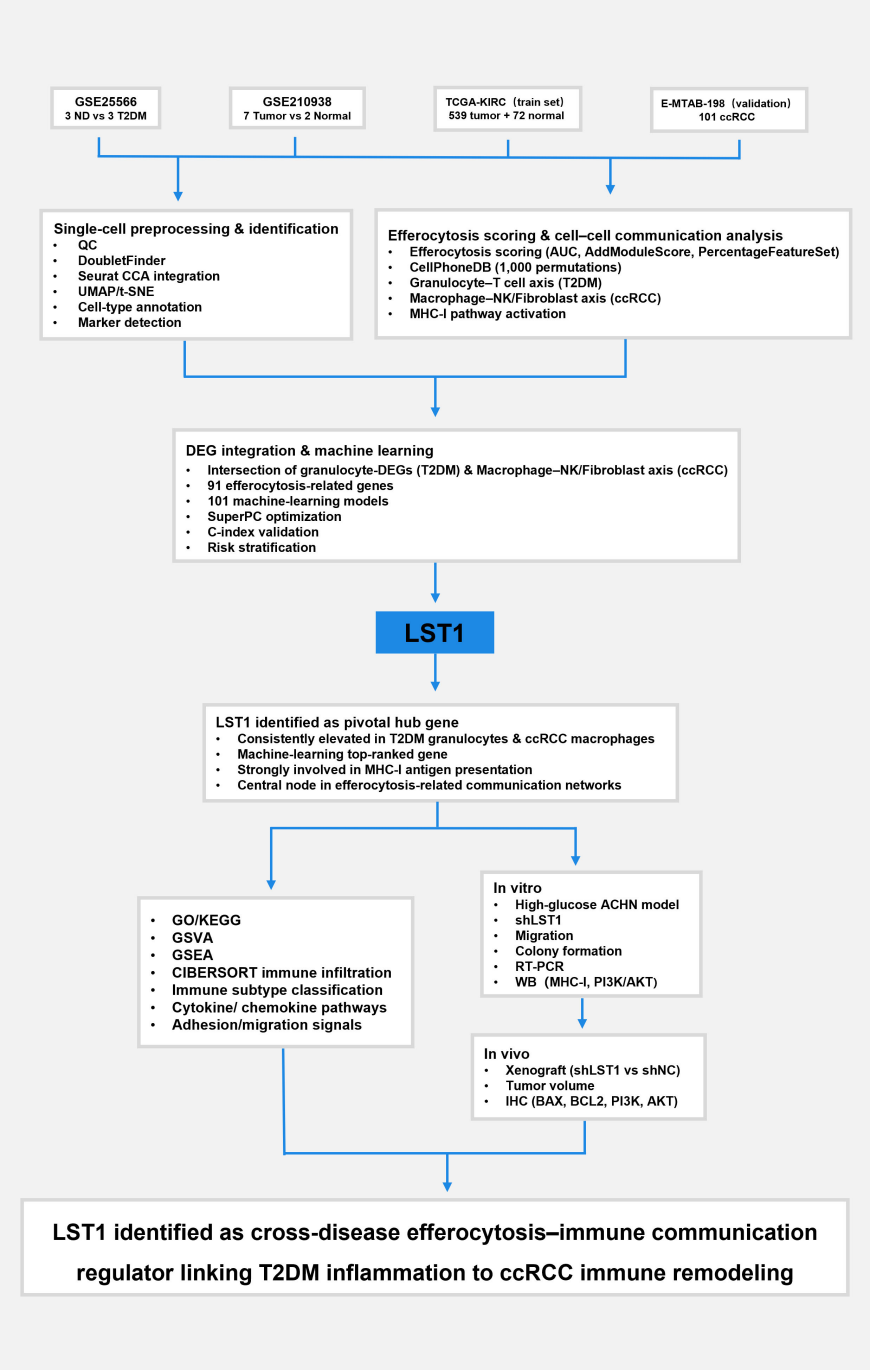
Study workflow illustrating the integrated single-cell, efferocytosis, machine-learning, and experimental validation pipeline leading to the identification of LST1 as the key cross-disease regulator. The diagram outlines data acquisition, preprocessing, communication analysis, DEG integration, hub-gene screening, immune profiling, and *in vitro*/*in vivo* functional assays.

We obtained data on T2DM and ccRCC from the Gene Expression Omnibus (GEO) database (hosted by the National Center for Biotechnology Information [NCBI]; https://www.ncbi.nlm.nih.gov/geo/) (14). The scRNA-seq datasets for ccRCC was derived from the GPL20301 Illumina HiSeq 400 human genome microarray study, comprising samples from 7 ccRCC patients and 2 non-ccRCC samples. The T2DM-related scRNA-seq dataset GSE255566 involved 3 non-diabetic individuals and 3 diabetic patients following the GPL29480 protocol. Transcriptome data for the training set were sourced from the KIRC dataset within the TCGA database, encompassing 539 ccRCC samples and 72 normal tissue samples. Data for the validation set, which included 101 ccRCC samples, were retrieved from E-MTAB-198 (OmicsDI: Home) (15–17). An overview of how these datasets were integrated and incorporated into the analytical pipeline is illustrated in **Figure 1**.

### Dimensionality reduction analysis and cell type identification

Doublet removal was performed using DoubletFinder (v2.0) with an expected doublet rate of 5% and default parameter optimization. Batch correction and data integration were conducted using the Seurat v4 CCA-based integration workflow, and integration quality was evaluated by UMAP-based mixing of samples, silhouette scores and the absence of batch-driven separation in PCA space. For marker detection, we adopted a relatively low threshold of |log2(Fold Change)| ≥ 0.1 in order to capture subtle but biologically meaningful transcriptional shifts in single-cell data; importantly, cluster definition was driven by the shared nearest-neighbor graph topology rather than DEG magnitude, so this threshold did not affect clustering stability.

We processed the initial gene expression matrix. Using Cell Ranger software (version 4.1.0.), we aligned reads to the reference genome (Homosapiens_GRCh38_96). Subsequently, Seurat package (version 4.3.0) in R (version 4.1.0) was employed for data filtering and normalization using the FindNeighbors and FindClusters functions. In the context of T2DM, cells were filtered based on the criteria: nFeature_RNA > 500 and < 10000, and percent.MT < 10. For T2DM samples, we filtered cells using the following criteria: number of detected features (nFeature_RNA) > 500 and < 10,000, and percentage of mitochondrial genes (percent.MT) < 10%. Subsequently, the ScaleData and RunPCA functions were applied for standardizing single-cell RNA data and conducting principal component analysis (PCA). Dimensionality reduction was performed using the RunTSNE and RunUMAP functions to implement t-SNE and UMAP algorithms. Marker genes were identified by comparing differentially expressed genes (DEGs) in each cluster against all other clusters using the FindMarkers function, with criteria set at |log_2_(Fold Change)| ≥.1, cell population expression proportion ≥ 0.25, and P-value < 0.05. Cell types were determined by comparing identified marker genes with known cell-type marker genes from the CellMarker database (18, 19).

### Intercellular communication and construction network

Information on intercellular communication was obtained from the CellPhoneDB database (https://www.cellphonedb.org/). Mean expression levels of ligands and receptors in specific cell types were determined based on the percentage of cells expressing specific genes and their average expression levels. Subsequently, cell-specific ligand-receptor pairs were identified. The protein-protein interaction network was visualized using the Cytoscape software (20, 21).

### Construction of PPI network and identification of core genes

STRING-derived protein–protein interaction networks were restricted to high-confidence edges (interaction score > 0.7), and hub modules were identified using CytoHubba (degree and MCC metrics) together with MCODE clustering (k-core = 2) in Cytoscape.

Investigating molecular targets, signaling pathways, metabolic pathways, and network functions related to efferocytosis can be enhanced by analyzing molecular interactions of comorbidities and protein-protein interaction (PPI) networks. Target genes were inputted into the STRING database (https://string-db.org/) for analysis, and the resulting PPI network was visualized utilizing Cytoscape software. Key genes within the constructed PPI network were identified using the CytoHubba and MCODE plugins within Cytoscape software (22).

### Machine learning analysis

By examining the overlap of differentially expressed genes in neutrophils from individuals with T2DM and macrophages in ccRCC pertaining to efferocytosis, we identified diagnostic differentially expressed genes for comorbidity analysis associated with efferocytosis. Ten machine learning algorithms, such as stepwise Cox regression, random survival forest (RSF), elastic net (Enet), supervised principal components (SuperPC), Cox partial least squares regression (plsRcox), CoxBoost, survival support vector machine (SVM), Lasso, Ridge, and generalized boosting regression model (GBM), were combined into 101 algorithmic configurations through 10-fold cross-validation in the TCGA-CRC cohort. The Harrell’s C-index value of the E-MTAB-198 cohort was computed to select the optimal risk prediction model. Subsequently, patients were stratified into high-risk and low-risk groups based on the median risk score of the cohort. Kaplan-Meier survival curve analysis was conducted using the survminer package in R to compare survival outcomes between the high-risk and low-risk groups, while the receiver operating characteristic (ROC) curve was utilized to evaluate the prognostic performance of the risk prediction model. Furthermore, two conventional machine learning approaches were employed to refine the selection of potential diagnostic genes. Specifically, LASSO regression analysis and random forest analysis were performed using the glmnet and randomForest packages, respectively. The overlapping genes were identified as the key genes for diagnosing the comorbidity of T2DM-ccRCC in the context of efferocytosis (23–25).

### Immune cell infiltration

A multidimensional bioinformatics approach was utilized to systematically analyze the molecular mechanisms underlying risk stratification. Initially, functional activity scores for landmark pathways were computed using the Gene Set Variation Analysis (GSVA) algorithm. Subsequently, the limma differential analysis toolkit was applied to compare pathway activity differences between high-risk and low-risk groups, with the aim of identifying key regulatory pathways closely associated with risk characteristics. To elucidate the biological underpinnings of risk classification further, Gene Set Enrichment Analysis (GSEA) was conducted using the clusterProfiler package in R language. Systematic annotation was performed on the three primary functional modules in the Gene Ontology (GO) database: Biological Processes (BP), Cellular Components (CC), and Molecular Functions (MF). A dual screening criterion was employed, requiring an absolute value threshold of the Normalized Enrichment Score (|NES| > 1) in conjunction with a False Discovery Rate (FDR < 0.25) to ensure both statistical significance and biological relevance of the identified differential functional modules (26).

To investigate the potential relationship between immune checkpoint-related risk scores and immune cell infiltration in the tumor microenvironment, a comprehensive computational approach was utilized for validation. Initially, the CIBERSORT algorithm was employed for deconvolution analysis of tumor tissue transcriptome data to quantitatively assess the abundance of 22 immune cell subsets. Additionally, the ESTIMATE algorithm was utilized to globally quantify the stromal and immune components of the tumor microenvironment to bolster the robustness of the findings. Furthermore, single-sample gene set enrichment analysis (ssGSEA) was integrated to analyze the expression patterns of immune cell-specific marker genes. By employing these three methodologies collaboratively, the study not only accurately quantified immune cell infiltration levels but also validated the research conclusions rigorously through various perspectives, including the dynamic equilibrium of stromal-immune components in the microenvironment and the consistency of gene expression profiles (27).

### Cell culture and transfection

Human renal cell adenocarcinoma cells (ACHN cells) and mouse macrophage cell line (RAW264.7) were procured from the Shanghai Branch of the Chinese Academy of Sciences and maintained in DMEM medium supplemented with 10% heat-inactivated FBS. The cells underwent treatment with either normal glucose (NG, 5.6 mM) or high glucose (HG, 30 mM) solutions for a duration of 72 hours. Cells were transfected with LST1-targeting short hairpin RNA (shLST1) using Lipofectamine 200 reagent (Invitrogen), following the manufacturer’s protocol. Subsequently, the cells were rinsed with serum-free Dulbecco’s Modified Eagle’s Medium (DMEM), suspended in Diluent C (Sigma-Aldrich) at a concentration of 2×10^7^ cells/mL, and exposed to 4 µL PKH26 dye (Sigma-Aldrich) for 2–3 minutes. The cells were then washed twice with heat-inactivated DMEM supplemented with 10% fetal bovine serum (FBS) (28).

### Colony formation and wound healing assays

High-glucose co-cultured cells were transfected with sh-NC, shLST1#1, and shLST1#2 lentiviruses, seeded into 6-well plates, and cultured until reaching sub-confluency. A linear scratch was created using a pipette tip, and images were captured at 0 and 24 hours post-scratching. The scratch area was measured in triplicate to assess the cell healing rate. Subsequently, 100 transfected high-glucose ACHN cells were seeded per well in 6-well plates and cultured in RPMI-164 medium supplemented with 10% fetal bovine serum for 10 days. The resulting colonies were fixed with methanol, stained with 1% crystal violet, and imaged.

### Real-time quantitative polymerase chain reaction

Total RNA was extracted from cells using Trizol reagent, followed by reverse transcription with M-MuLV reverse transcriptase from Sangon Biotech (Shanghai) to synthesize cDNA. PCR amplification of the target genes was conducted using the SYBR Green real-time quantitative PCR system (StepOne Real-Time PCR System) from Sangon Biotech (Shanghai) and the StepOne Software v2.3 on a quantitative PCR instrument from ABI (USA). The primer sequences for the target genes were as follows: LST1 forward primer, TCCTCAGAGCAGGAACTCCA, and reverse primer, GCAATGCAGGCATAGTCAGC; GAPDH forward primer, GAGAAGGCTGGGGCTCATTT, and reverse primer, AGTGATGGCATGGACTGTGG. The CT values of the target genes and the reference gene GAPDH were documented. The 2^−ΔΔCt^ method was employed for semi-quantitative assessment of the mRNA expression levels of the target genes (29).

### Western blot

ACHN cells were harvested and lysed using sample buffer. Subsequently, protein denaturation was carried out, followed by SDS-PAGE on a 15% gradient Bis-Tris gel. The membrane was then blocked with 5% bovine serum albumin at 37°C for 2 hours, incubated with the primary antibody overnight at 4°C, and subsequently with the secondary antibody for 1 hour. Detection was accomplished through chemiluminescence, followed by protein expression analysis using a quantitative gel analysis system after three washes. ECL (Solarbio, Beijing, China) was utilized for detection, with GAPDH serving as the reference control (30).

### Nude mouse xenograft model

In the xenograft experiment, tumors derived from shLST1-transfected cells were directly compared with matched sh-NC control xenografts to isolate the functional contribution of LST1 silencing. Ten-week-old male Balb/c nude mice were housed in specific pathogen-free (SPF) conditions. Mice were randomly allocated into two experimental cohorts: the MOD group and the shLST1 group. Co-cultured cells, along with co-cultured cells stably transfected with the shLST1 plasmid, were suspended in a mixture of phosphate-buffered saline and Matrigel matrix to generate the respective cell suspensions. Subsequently, 4 × 10^6 cells were subcutaneously inoculated into each mouse. Tumor progression was assessed by monitoring tumor dimensions and mouse body weight. Tumor volume (mm^3) was determined using the standard formula: (width^2×length)/2. All experimental procedures were conducted in accordance with the guidelines approved by the Medical Ethics Committee of Nantong University (Approval No. S20250210-014).

### Immunohistochemical analysis

Tumor samples underwent immunohistochemical analysis following standard histological section staining procedures (10% neutral buffered formalin-fixed, paraffin-embedded, and sectioned). Antibodies against BAX (batch no. CAT5023S), BCL-2 (batch no. CAT15071S), PI3K (batch no. CAT19591S), and AKT (batch no. CAT4691S) were employed. Image J was utilized for data analysis.

### Statistical analysis

Data analysis was conducted using R 4.1.0 software and GraphPad Prism 10. The Wilcoxon rank-sum test compared two groups, while the Kruskal–Wallis test compared more than two groups. Survival analysis utilized the Kaplan–Meier method with the log-rank test. Pearson correlation analysis was employed for key genes. Statistical significance was defined as *p* < 0.05.

## Results

### Identify the gene expression profiles in T2DM

**Figure 2:**
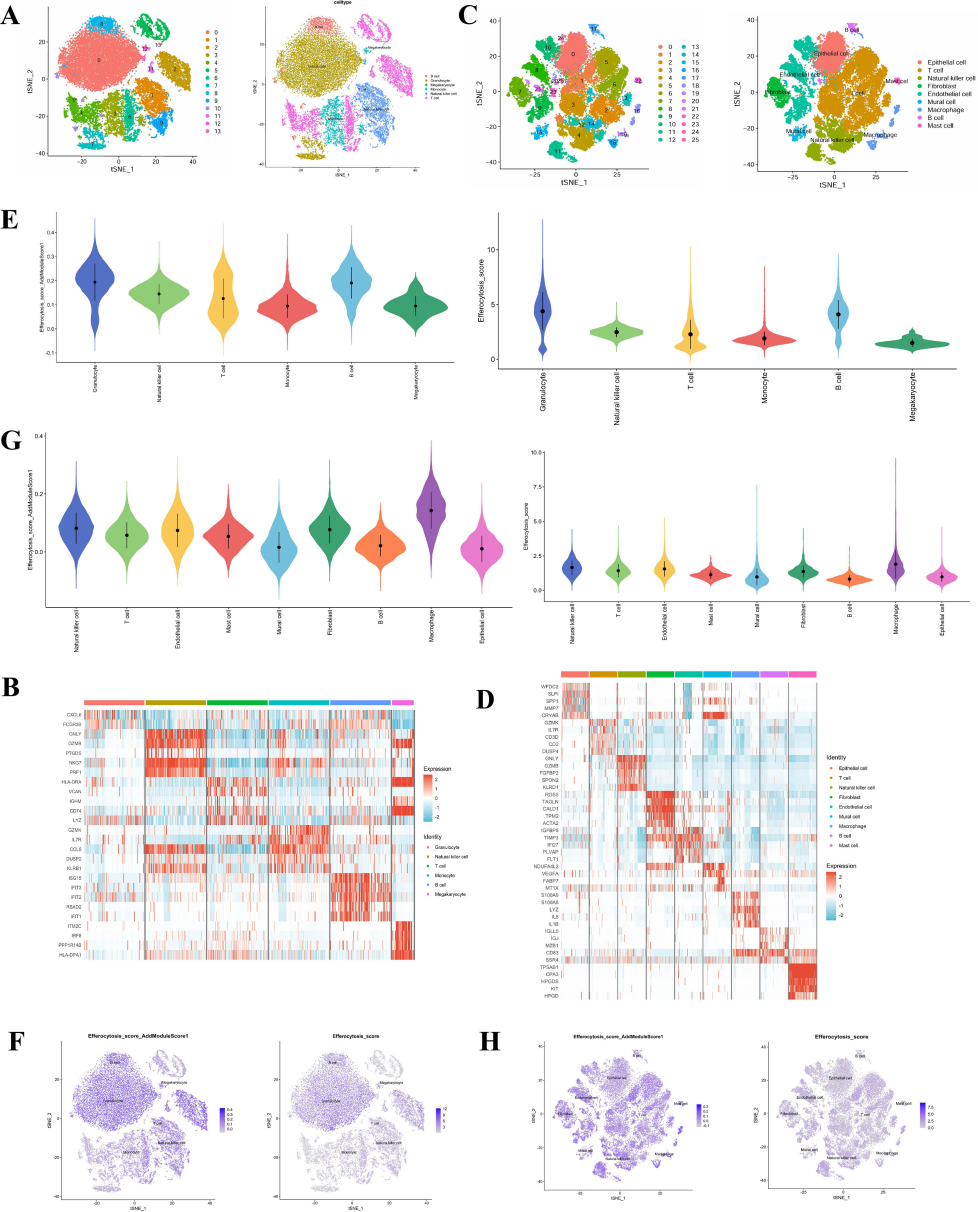
The tSNE clustering plot and cell type annotation for the T2DM dataset **(A)**, a heatmap illustrating the expression of characteristic genes in cell subsets of the T2DM dataset **(B)**, the tSNE clustering plot and cell type annotation for the ccRCC dataset **(C)**, a heatmap showing the expression of characteristic genes in cell subsets of the ccRCC dataset **(D)**, the distribution of efferocytosis scores across different cell types in the T2DM data **(E)**, a two-dimensional tSNE spatial representation of efferocytosis scores in the T2DM data **(F)**, the distribution of efferocytosis scores for various cell types in the ccRCC data **(G)**, and a two-dimensional tSNE spatial distribution of efferocytosis scores in the ccRCC data **(H)**.

scRNA-seq data from T2DM samples in the GSE255566 dataset were log-normalized and analyzed for integration quality and batch effects. The six samples exhibited good integration without noticeable batch effects, ensuring their suitability for subsequent analysis. Unsupervised clustering analysis on the filtered cells was conducted using the “FindNeighbors” and “FindClusters” functions in the “Seurat” package, with a resolution parameter ranging from 0.01 to 3. PCA was employed for dimensionality reduction, selecting principal components with *p* < 0.05 for further analysis. We identified a total of 14 cell clusters, which were visualized using a t-distributed stochastic neighbor embedding (t-SNE) plot. Cell clusters were annotated using the Single R algorithm with reference to known cell type marker genes from the CellMarker database, revealing six cell types: granulocytes, monocytes, T cells, B cells, natural killer cells, and megakaryocytes (**Figure 2A**). Specifically, a heatmap showed that signature genes (e.g., CXCL8, FCGR3B, CD79A, GZMB) were highly expressed across these cell types, confirming the accuracy of cell annotation (**Figure 2B**).

### Identify the gene expression profiles in ccRCC

The scRNA-seq data for ccRCC in this study were obtained from tumor samples in the GSE210038 dataset. Following logarithmic normalization, the single-cell sequencing data underwent analysis, revealing good integration among the 9 samples without noticeable batch effects, rendering them suitable for subsequent analysis. Unsupervised clustering analysis on the filtered cells was conducted using the “FindNeighbors” and “FindClusters” functions within the “Seurat” package, with resolutions ranging from 0.01 to 3. PCA was employed for dimensionality reduction, with selection of principal components having p-values below 0.05 for further analysis. A total of 26 clusters were identified and visualized through t-SNE plots. Subsequently, leveraging known cell-type marker genes from the CellMarker database, the Single R algorithm was utilized to annotate these cell clusters, resulting in the identification of 9 cell types, including epithelial cells, endothelial cells, fibroblasts, mural cells, macrophages, T cells, B cells, and mast cells (**Figure 2C**). The corresponding heatmap depicted the high-expression profiles of various cell types in marker genes such as KRT19, VWF, ACTA2, CD68, and TPSB2, effectively distinguishing immune, stromal, and tumor cell components (**Figure 2D**). Notably, macrophages were significantly enriched in ccRCC tissues and expressed multiple key factors involved in immune regulation and phagocytosis-related pathways. This finding suggests that macrophages play a pivotal role in shaping the tumor immune microenvironment (TIME) of ccRCC. Given the significant enrichment of macrophages in ccRCC tissues and their high expression of key factors involved in immune regulation and phagocytosis-related pathways, we further investigated the functional role of macrophages in efferocytosis, which is a core process of phagocytic clearance of apoptotic cells and closely associated with tumor immune microenvironment remodeling.

### Distribution characteristics in dominant efferocytosis in T2DM and ccRCC

Based on the aforementioned cell composition analysis of T2DM and ccRCC, we employed two scoring methods (AddModuleScore and PercentageFeatureSet) using a well-annotated efferocytosis-related gene set to evaluate efferocytosis activity across all cell types. This analysis aimed to identify the functional status of efferocytosis and the pivotal cell populations mediating this process in both diseases, thereby providing a cellular basis for understanding T2DM-ccRCC comorbidity. In the T2DM dataset, utilizing the AddModuleScore method revealed a notably higher efferocytosis score in the granulocyte subset compared to other cell types, a trend consistently supported by the PercentageFeatureSet score (**Figure 2E**). Visualization through a two-dimensional tSNE projection indicated that cells exhibiting elevated efferocytosis scores were predominantly situated within the granulocyte cluster (**Figure 2F**), suggesting that granulocytes may predominantly engage in the clearance of deceased cells in the context of T2DM, displaying heightened efferocytosis activity. Similarly, within the ccRCC dataset, the efferocytosis scoring outcomes demonstrated significantly higher scores in the macrophage population using both AddModuleScore and PercentageFeatureSet compared to other cell types (**Figure 2G**). The spatial distribution analysis further confirmed that cell populations with elevated efferocytosis scores were predominantly localized within the macrophage cluster (**Figure 2H**), indicating that macrophages likely govern the regulatory mechanisms of efferocytosis within the tumor immune microenvironment, playing a crucial role in antigen presentation and inflammation modulation.

### Cellular communication networks and shared pathways

CellPhoneDB analyses were performed with 1,000 permutations, and ligand–receptor pairs with permutation-based p values < 0.05 were considered significant to ensure robustness of inferred cell–cell communications. To investigate intercellular signal communication characteristics and potential shared mechanisms in T2DM and ccRCC disease models, a cell communication network was developed using single-cell data. Signal input and output intensities, as well as pathway distributions among key cell subsets, were compared across different disease contexts.

**Figure 3:**
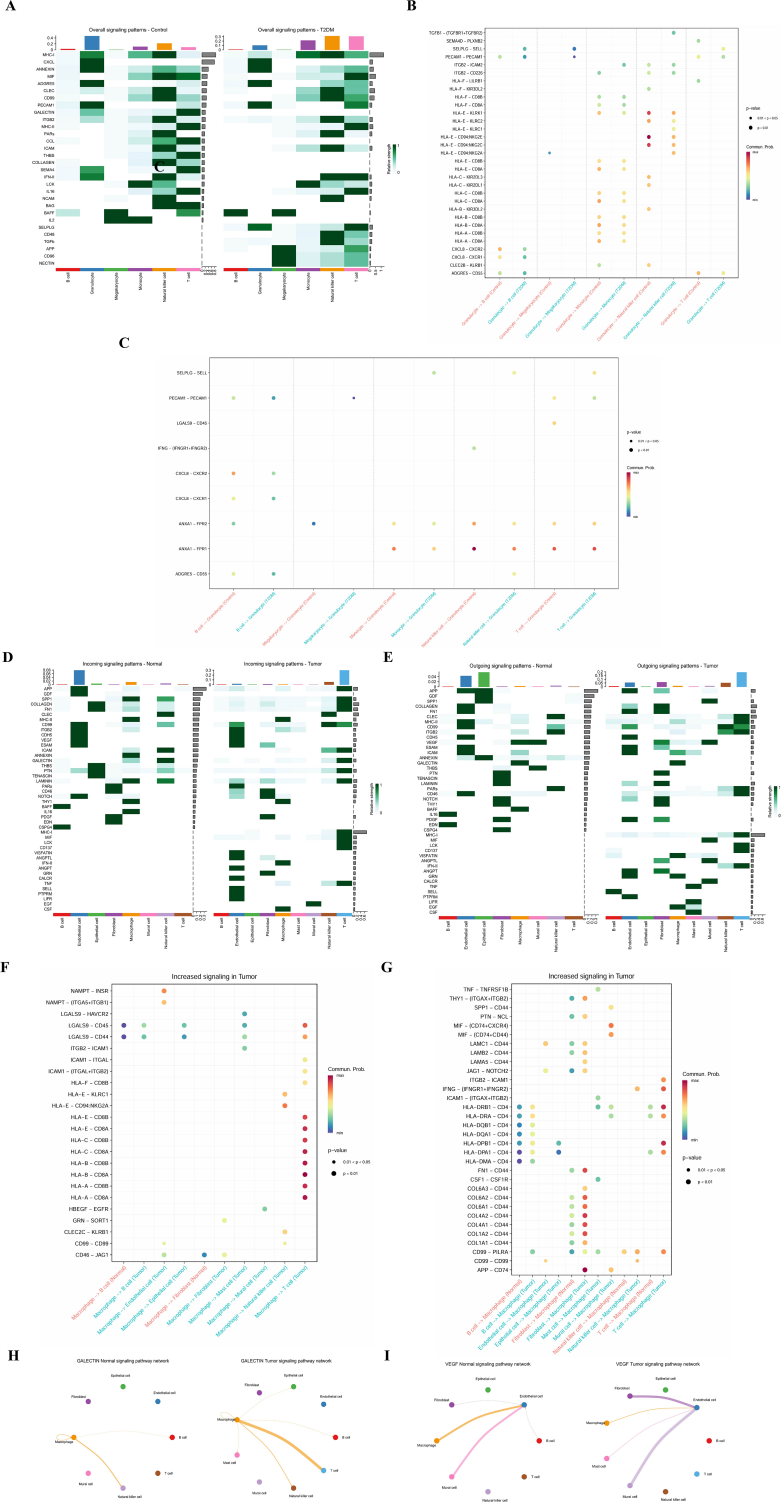
A heatmap illustrating the signal intensity differences of various cell types between the normal and T2DM groups **(A)**, scatter plots depicting the primary ligand-receptor interactions among cell types in the normal and T2DM groups **(B, C)**, signal reception intensity mapping comparing the normal and tumor groups in ccRCC **(D)**, signal output intensity mapping comparing the normal and tumor groups in ccRCC **(E)**, identification of significantly heightened signaling pathways in the ccRCC tumor group **(F, G)**, and signal network diagrams of the GALECTIN and VEGF pathways in normal and tumor states **(H, I)**.

In the T2DM dataset, the global signal intensity mapping reveals a notable increase in granulocyte signal output activity in the diseased state, particularly in pathways including CXCL, ADGRE5, PECAM1, and MIF (**Figure 3A**). Comparative analysis between the T2DM condition and normal controls indicates heightened interactions of granulocytes with monocytes, T cells, and mast cells through diverse chemokine and immune regulatory pathways (e.g., HLA-A/B/C/E, SELP–SELL), underscoring their pivotal role in inflammation modulation and immune cell recruitment (**Figures 3B, C**). Moreover, the elevated expression levels of signal pairs such as ANXA1-FPR1/2 and PECAM1-PECAM1 suggest potential involvement of granulocytes in intercellular adhesion and phagocytosis.

In the ccRCC dataset, macrophages demonstrated robust signaling capabilities within the tumor microenvironment, engaging in various pathways including COLLAGEN, APP, and CD96 (**Figures 3D, E**). Compared to normal tissues, macrophage communication with fibroblasts, T cells, and endothelial cells was notably heightened in tumor tissues. Notably enriched signal pairs such as ICAM1-ITGB2, MIF-CD74, COL1A2-CD44, and HLA-A/B/C/E indicated potential involvement in processes such as antigen presentation, cell recruitment, and immune evasion by tumor cells within the tumor microenvironment (**Figures 3F, G**). Subsequent pathway network analyses highlighted the significance of the GALECTIN and VEGF pathways in different disease states. In ccRCC, the GALECTIN pathway primarily emanated from macrophages, with notable shifts in target cell types between normal and diseased states, suggesting a context-dependent mode of communication (**Figure 3H**). The VEGF signaling network exhibited a pronounced preference for endothelial cells in tumor tissues, implying its potential role in critical processes like angiogenesis and extracellular matrix remodeling during ccRCC progression (**Figure 3I**).

In summary, both the T2DM and ccRCC models demonstrate a predominant signaling communication pattern involving granulocytes and macrophages, with shared pathways indicating a potential bridging role in the comorbidity mechanism of these two diseases.

### Develop a predictive model and assess its prognostic efficacy

**Figure 4:**
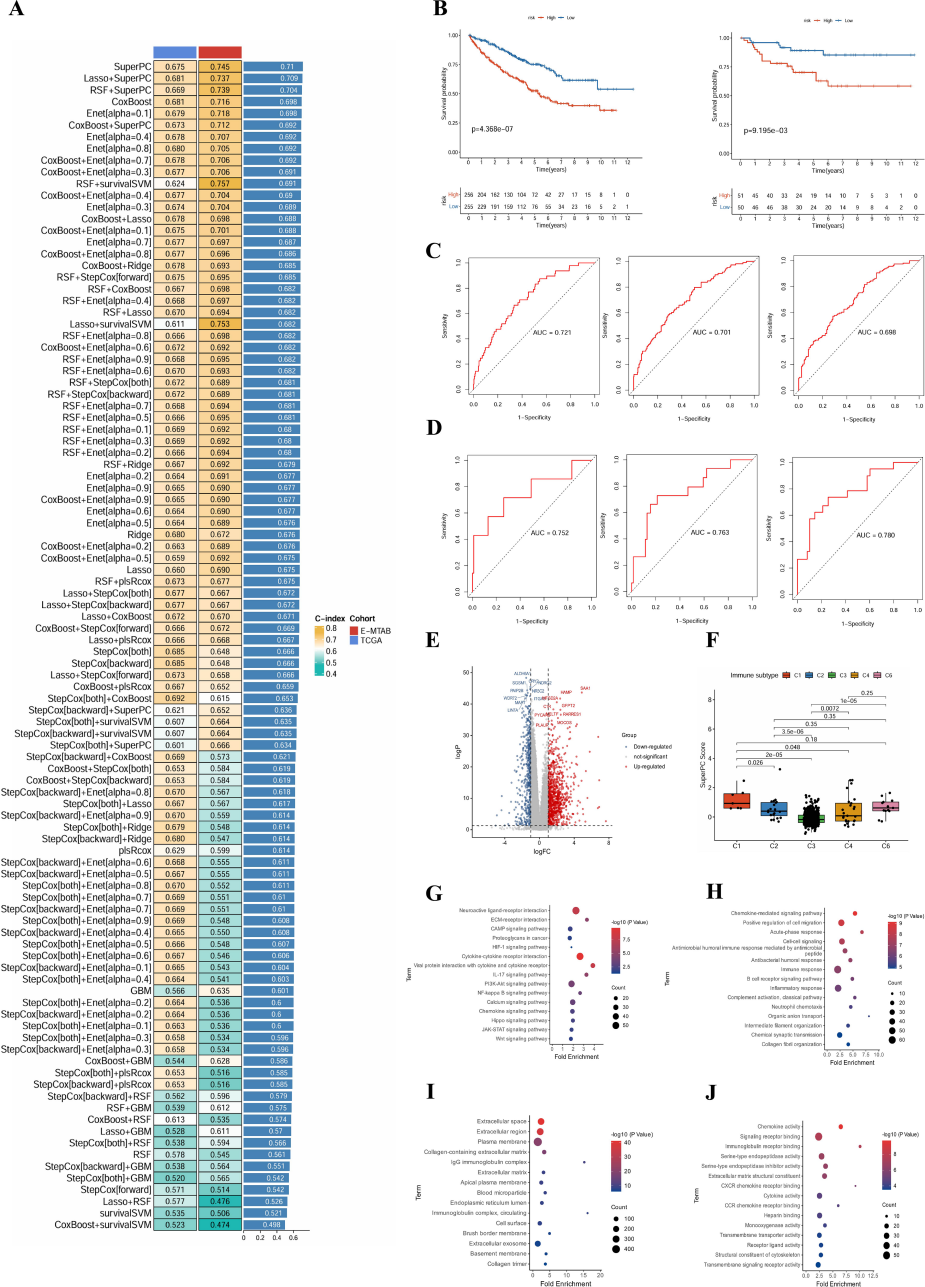
C-index heatmap evaluating the performance of 101 combined machine learning models **(A)**. Kaplan-Meier survival curves demonstrating the prognostic discrimination ability of the SuperPC score grouping in the TCGA training set and the E-MTAB-198 validation set, respectively **(B)**. ROC curves for 1-year, 3-year, and 5-year in the TCGA training set and the corresponding curves in the validation set E-MTAB-198 **(C, D)**. Volcano plot indicating the distribution of differentially expressed genes between the SuperPC high and low score groups **(E)**. Distribution variances of SuperPC scores among different immune subtypes **(F)**. Results of KEGG pathway enrichment **(G)**. Biological processes, cellular components, and molecular functions for the differential genes **(H-J)**.

Combining the differential efferocytosis activity of granulocytes in T2DM and macrophages in ccRCC, as well as the shared signaling pathways (e.g., HLA, CXCL pathways) enriched in the intercellular communication network, we performed intersection analysis of differentially expressed genes (DEGs) from these two core cell populations. This analysis yielded 91 potential candidate genes associated with T2DM-ccRCC comorbidity. To identify core diagnostic factors underlying the comorbidity mechanism, we systematically evaluated 101 combinations of machine learning models, including 10 algorithms such as Cox regression, LASSO, and random survival forest (RSF) (**Figure 4A**). The SuperPC model exhibited the highest performance based on the comprehensive C-index score in the TCGA-KIRC training cohort (C-index = 0.745) and demonstrated stability in the E-MTAB-198 validation set, indicating robust generalization ability. Consequently, the SuperPC model was chosen as the optimal model, dividing patients into high-risk and low-risk groups according to the model’s median score. Kaplan-Meier survival analysis showed that the high-risk group had a significantly lower overall survival (OS) rate than the low-risk group (**Figure 3B**; TCGA cohort: p = 3.88×10^−7^; validation set [E-MTAB-198]: p = 1.95×10^−^³). In terms of prognostic performance evaluation, the areas under the receiver operating characteristic (ROC) curves (AUC) for 1-year, 3-year, and 5-year predictions in the TCGA training set were 0.721, 0.701, and 0.780, respectively (**Figure 4C**). Corresponding AUC values in the E-MTAB-198 validation set were 0.752, 0.763, and 0.698, respectively (**Figure 4D**), demonstrating strong time-dependent predictive capability. These findings suggest that the SuperPC model, constructed based on efferocytosis-related comorbidity differential genes, not only exhibits significant prognostic discriminatory power but also displays cross-cohort stability, supporting its utility as a tool for comorbidity mechanism investigation and potential clinical early warning systems.

### SuperPC model reveals molecular pathways and immune infiltration

To elucidate the biological implications conveyed by the SuperPC model score, the TCGA-KIRC samples were stratified into high-score and low-score cohorts utilizing the median of the model score as the delineation point. Subsequent analyses encompassed differential expression, functional enrichment, and characterization of the immune microenvironment features.

Differential expression analysis revealed a substantial number of significantly differentially expressed genes between high- and low-score groups (**Figure 4E**). Notably, genes like SAA1, PYCR1, PURA, and RAPGEF5 were significantly upregulated in the high-score group, whereas ALDH4A1 and WDR72 showed higher expression in the low-score group. This differential expression pattern suggests that the SuperPC score not only serves as a prognostic predictor but also reflects the molecular and immune characteristics of tumor tissues. To delve deeper into the biological implications of the SuperPC score, we conducted a correlation analysis between the score and the classic TCGA immune subtypes. Our findings demonstrated a significant gradient distribution of the score across different immune subtypes: C2 (IFN-γ dominant subtype) and C3 (inflammatory subtype) exhibited the lowest scores and the most favorable prognosis, indicative of a robust anti-tumor immune microenvironment. In contrast, C1 (wound healing subtype) and C4 (lymphocyte depletion subtype) displayed the highest scores and the poorest prognosis, representing an immunosuppressive microenvironment. Notably, statistical tests confirmed significant score differences between C1, C4, and C2, C3 subtypes (e.g., C1 *vs* C2, *p* = 0.026; C4 *vs* C3, *p* = 3.5e^-06^). A low SuperPC score signifies a microenvironment characterized by highly active cellular immunity or a strong inflammatory background (C2, C3), associated with a relatively favorable prognosis. Conversely, a high score indicates an immune microenvironment with suppressed or exhausted functions (C1, C4), linked to a poor prognosis. Intriguingly, our model classified the typically considered “poor” inflammatory C3 subtype and the “good” C2 subtype into the low-risk group, suggesting that any form of immune infiltration or inflammation may imply a relatively better outcome compared to an “immune desert” scenario (such as C5), with the actual risk stemming from immune suppression (**Figure 4F**).

Furthermore, GO and KEGG enrichment analyses unveiled functional disparities among genes linked to high- and low-score groups. KEGG analysis (**Figure 4G**) highlighted significant enrichment of differentially expressed genes in critical immune and inflammatory pathways like ECM-receptor interaction, PI3K-Akt, Cytokine–cytokine receptor interaction, IL-17, and NF-κB. GO biological process analysis (**Figure 4H**) indicated close associations of these genes with processes such as B cell-mediated immunity, complement activation, neutrophil chemotaxis, and chemokine signal transduction. Cellular component annotation (**Figure 4I**) revealed widespread gene localization in extracellular matrix, immunoglobulin complexes, plasma membranes, and exosomes. Molecular function analysis (**Figure 4J**) further underscored functional attributes like chemokine activity, cytokine binding, and signal receptor ligand activity, emphasizing their pivotal role in immune cell communication and regulation.

**Figure 5:**
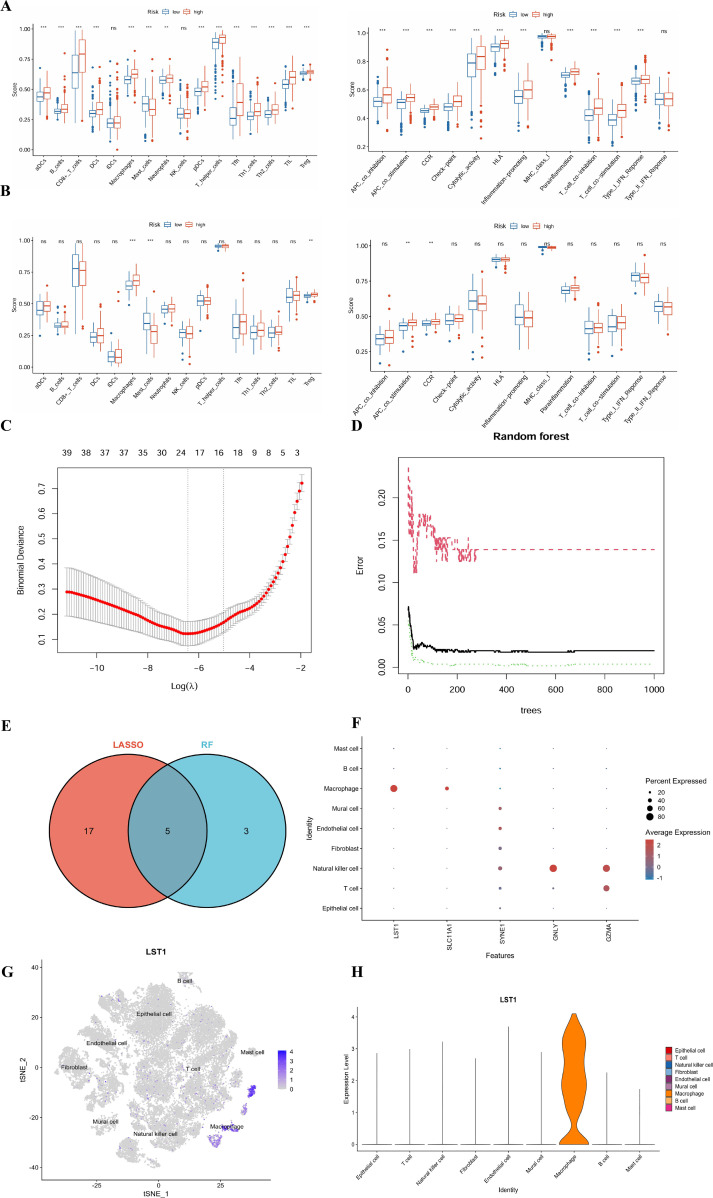
Scores of immune cell infiltration characteristics and immune function items using ssGSEA in the TCGA-KIRC cohort **(A)**. Distribution of immune cell infiltration and immune function status in the high/low score groups of the E-MTAB-198 validation set **(B)**. LASSO regression analysis **(C)**. Error curve of feature genes ranked by importance in the random forest analysis **(D)**. Five key genes derived from the intersection of LASSO and RF methods **(E)**. Expression proportion and average expression level of these five intersecting genes in each cell type in the single-cell data by a DotPlot **(F)**. tSNE expression distribution of LST1 in different cell clusters in a FeaturePlot **(G)**. Expression level distribution of LST1 in various cell types in a Violin plot **(H)**. Note: * represents for vs NC group (p < 0.05), ** represents for vs NC group (p < 0.01), *** represents for vs NC group (p < 0.001), respectively.

Based on the identified differentially expressed genes, we utilized the ssGSEA method to evaluate immune infiltration patterns and immune function scores in samples categorized into high- and low-score groups. Within the TCGA cohort, the high-score group demonstrated elevated levels of immune cell infiltration, notably CD8^+^ T cells, Th1 cells, M1 macrophages, and NK cells, indicative of heightened immune activation. Moreover, this group exhibited increased expression of immune function modules such as APC co-stimulation, cytotoxicity, inflammation promotion, and HLA (**Figure 5A**). The substantial immune cell infiltration depicted suggests a highly activated tumor microenvironment in the high-score group, albeit accompanied by robust immune checkpoint signals, characteristic of a ‘functionally exhausted’ phenotype. This phenomenon elucidates the unfavorable prognosis observed in patients despite a vigorous immune response and suggests that individuals within this subgroup may benefit from immune checkpoint inhibitor therapy.

To validate the generalizability of these observations, a parallel analysis was conducted in the independent E-MTAB-198 cohort. The results mirrored the trends observed in the TCGA training set, demonstrating consistent patterns of immune infiltration and functional activity between the high- and low-risk groups (**Figure 5B**). While statistical significance in the infiltration levels of certain immune cell subtypes was not achieved due to the smaller sample size of the validation set, a notable upregulation of immune function modules, particularly cytotoxic activity and checkpoint signals, was faithfully replicated in the high-risk group. This inter-cohort reproducibility strongly supports the robust association between the SuperPC score and the ‘functionally exhausted’ immune microenvironment phenotype, underscoring its reliability beyond specific datasets. This reaffirms the potential of this model as a valuable tool for evaluating immune microenvironment status across diverse cancer types.

In conclusion, the SuperPC model demonstrates exceptional prognostic discrimination ability, indicating a high-risk state characterized by robust immune infiltration and functional activation. These findings suggest that the model may represent a ccRCC subtype with pronounced immunological activation, laying a robust groundwork for further investigation into the associated immune mechanisms.

### LST1 as a pivotal candidate gene for diagnosing comorbidities

To systematically identify key regulatory factors associated with the comorbidity of T2DM and ccRCC, we utilized the LASSO regression model for feature selection. Employing ten-fold cross-validation, it observed a gradual convergence of model error as the penalty parameter λ varied, leading to the identification of 24 candidate genes (**Figure 5C**). Subsequently, it assessed gene importance using the random forest algorithm, which revealed the stable behavior of model error with an increasing cumulative number of trees and identified 8 genes with significant contributions to classification (**Figure 5D**). By integrating the outcomes of both algorithms through Venn analysis, we identified 5 intersecting genes (**Figure 5E**). Further validation at the single-cell level demonstrated specific expression of LST1 in the immune cell population, particularly enriched in macrophages (**Figure 5F**). Visualization of t-SNE dimensionality reduction results indicated predominant localization of LST1 in the macrophage cluster (**Figure 5G**), aligning well with its putative roles in efferocytosis and immune regulation. This localization was further supported by the violin plot, confirming significantly elevated expression of LST1 in macrophages (**Figure 5H**). In summary, the combined findings from machine learning and single-cell analysis endorse LST1 as a pivotal candidate gene for comorbidity diagnosis, suggesting its potential key role in the progression of T2DM-ccRCC comorbidity through the regulation of macrophage-mediated efferocytosis and immune signaling.

### Remodeling of cell communication through LST1

**Figure 6:**
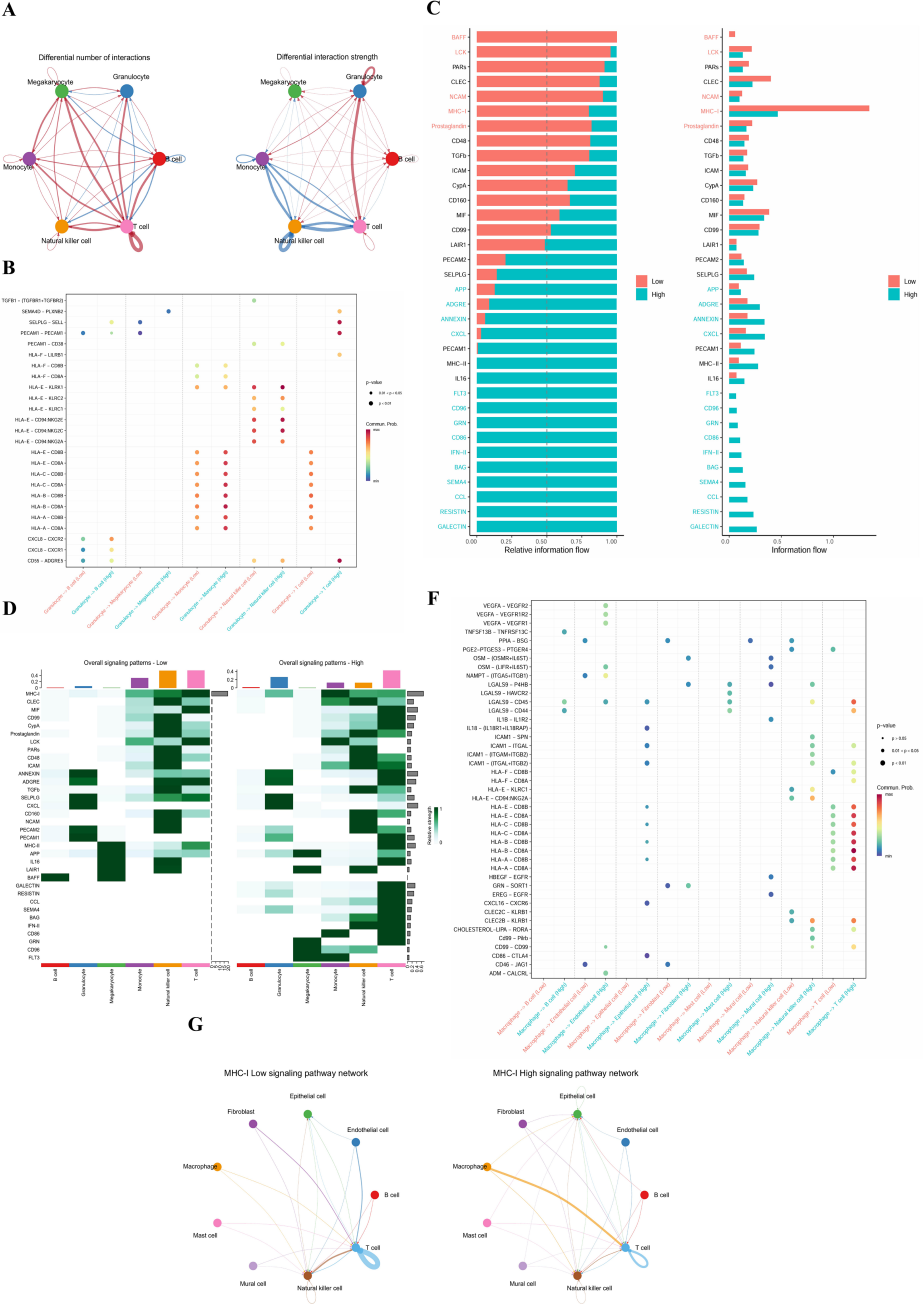
Signaling network diagram of T2DM **(A)**. Scatter plot showing the main ligand-receptor interactions between cell types in control and T2DM groups **(B)**. A bar chart indicating the communication flow between cell types in these groups **(C)**. A signal reception intensity map comparing normal and tumor groups in ccRCC **(D)**. a bar chart illustrating the communication flow between cell types in control and ccRCC groups **(E)**. Signaling network diagram of the MHC-1 pathway under normal and tumor conditions **(F)**.

Given the specific high expression of LST1 in macrophages (**Figure 5F**) and its strong correlation with efferocytosis and immune regulation functions (**Figures 5G, H**), we further explored the regulatory role of LST1 in the intercellular communication networks of T2DM and ccRCC. To clarify the immunological mechanism linking T2DM and ccRCC in the context of comorbidity, we stratified the T2DM and ccRCC datasets into high- and low-LST1 expression groups and systematically analyzed the differences in cell-cell communication patterns. In individuals with T2DM, granulocytes exhibiting high expression levels of LST1 demonstrate heightened immune communication characteristics. Specifically, their interactions with monocytes, T cells, and NK cells show both increased quantity and intensity (**Figure 6A**). Analysis using ligand-receptor dot plots further indicates elevated communication probabilities of signal pairs involved in antigen presentation and inflammatory responses, such as PECAM1-PECAM1, CXCL8-CXCR1, and HLA-E-CD94, within the high expression group. This suggests that granulocytes may augment their regulatory capacity within the local immune microenvironment through MHC molecule and chemokine pathways in the context of high LST1 expression (**Figure 6B**). The pathway information flow map corresponding to this reveal heightened levels of various immune signals, including MHC-I, TGF-β, and CD48. Notably, the communication flow of MHC-I between granulocytes and T cells is significantly increased (**Figure 6C**), indicating its pivotal role in LST1-mediated adaptive immunity regulation in T2DM.

In ccRCC, macrophages emerge as the primary mediators of immune signaling in the high LST1 expression cohort, amplifying their signaling capacity toward various cell types including T cells, fibroblasts, and natural killer cells (**Figure 6D**). The augmented presence of immune ligand-receptor pairs such as IL-6, TNFSF13B, LGALS9-CD45, and HLA-E-KIR (**Figure 6E**) underscores the pivotal role of macrophages in modulating inflammatory responses, immune evasion, and reshaping the tumor microenvironment within the context of elevated LST1 expression. Furthermore, a notable upregulation of MHC-I signals is evident in multiple cellular interactions (**Figure 6F**), indicating a pattern of immune communication restructuring akin to that observed in T2DM.

In essence, LST1 significantly activates the MHC-I pathway and its associated immune responses through immune communication networks mediated by granulocytes (in T2DM) and macrophages (in ccRCC), respectively. Western blot analysis further confirmed that knockdown of LST1 led to a significant decrease in MHC-I protein expression in ACHN cells (**Figures 6F, G**, p<0.01), suggesting that LST1 may directly regulate the transcription or translation of MHC-I pathway-related genes, thereby reshaping intercellular communication patterns in T2DM and ccRCC. This implies the existence of a shared immune regulatory axis centered on LST1-MHC-I in both diseases.

### Experimental verification of LST1 *in vitro* and *in vivo*

**Figure 7:**
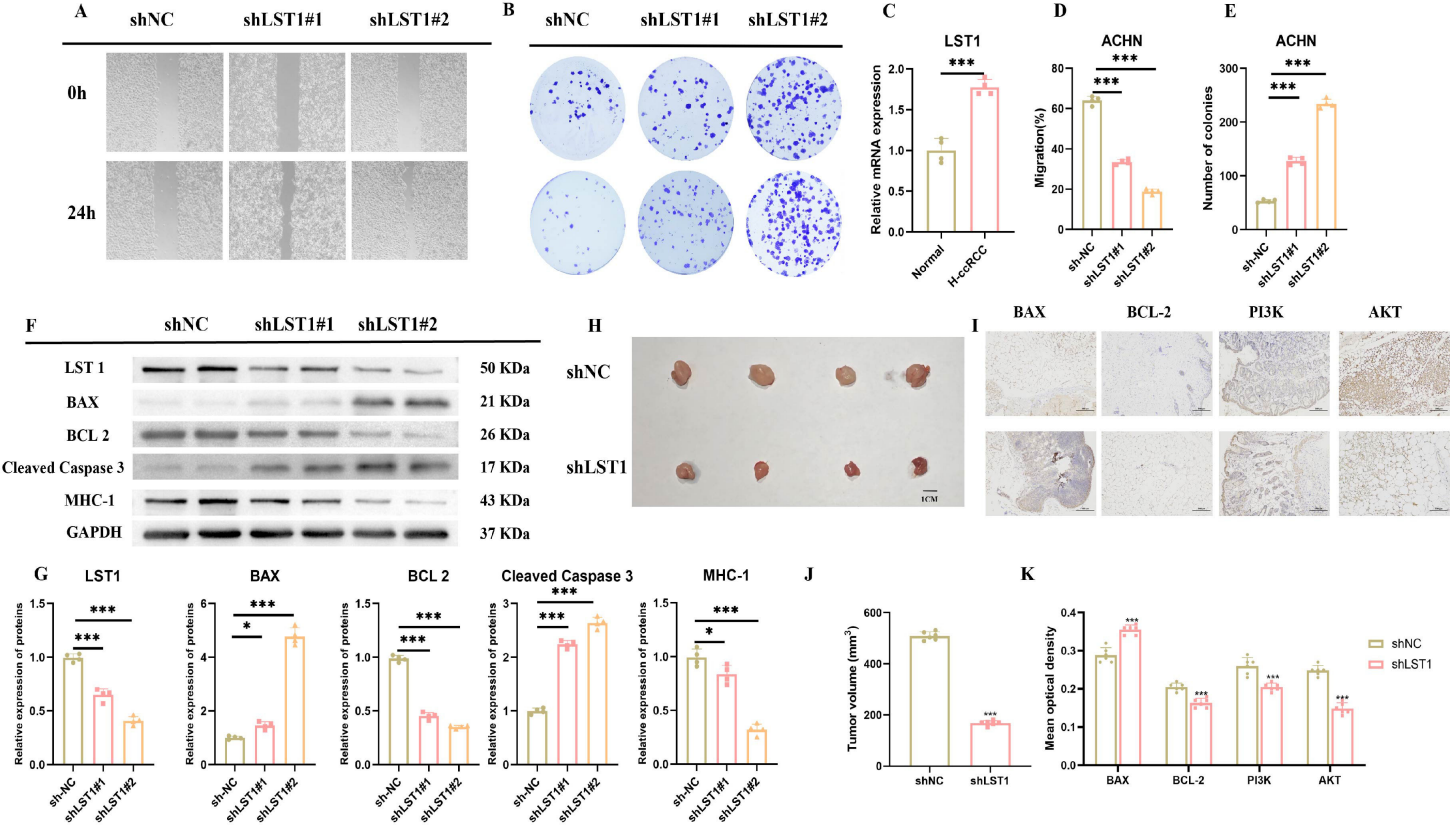
Outcomes of the wound scratch assay **(A)**. colony formation assay **(B)**. LST1 mRNA expression levels depicted in a histogram **(C)**. Migration efficiency in the scratch assay illustrated in a histogram **(D)**. Statistics on colony numbers represented in a histogram **(E)**. Western blot findings **(F)**. Histogram displaying the results of the Western blot analysis **(G)**. Images of tumor **(H)**. Immunoblotting plots at different solubilities **(I)**. Histogram displaying the results of Tumor volume **(J)**. Histogram of immunoblotting results **(K)**. Note: * represents for *vs* NC group (*p* < 0.05), ** represents for *vs* NC group (*p* < 0.01), *** represents for *vs* NC group (*p* < 0.001), respectively.

The study revealed that LST1 expression in ACHN cells exposed to high-glucose conditions was markedly elevated compared to normal cells, suggesting a pivotal role of LST1 in comorbidity progression (**Figure 7C**). Subsequent investigations into the functional significance of LST1 in high-glucose-induced ccRCC progression included colony formation and wound healing assays. Two shRNA plasmids targeting LST1 (shLST1#1 and shLST1#2) were constructed and transfected into high-glucose-cultured ACHN cells, resulting in a significant reduction in LST1 expression compared to the shNC control group (**Figure 7C**, p<0.001). Functional assays showed that shLST1-transfected cells exhibited significantly diminished migratory capacity (**Figures 7A, D**, p<0.01) and markedly reduced colony-forming ability (**Figures 7B, E**, p<0.001), consistent with the hypothesis that LST1 promotes ccRCC progression under high-glucose conditions. Western blot analysis confirmed effective transfection and revealed enhanced apoptosis and suppressed MHC-1 pathway in renal cancer cells (**Figures 7F, G**). Overall, LST1 activation was implicated in T2DM and ccRCC comorbidity. Knockout of LST1 led to substantial alterations in cell migration, colony formation, apoptosis, and MHC-1 signaling, underscoring LST1’s potential as a signaling marker and offering novel insights for comorbidity prevention and diagnosis. Notably, 21 days post shLST1 treatment, a substantial reduction in tumor volume was observed in the shLST1-treated group, underscoring the superior *in vivo* anti-proliferative efficacy, consistent with the findings from the *in vitro* assays (**Figures 7H, J**). The results revealed that LST1 knockdown significantly increased the expression of the pro-apoptotic protein BAX and concurrently decreased the anti-apoptotic protein BCL-2. Additionally, the expression of PI3K and AKT, key components of the pathway, was significantly decreased. These findings suggest that LST1 knockdown induces apoptosis in renal cancer cells, potentially through the PI3K/AKT pathway (**Figures 7I, K**).

## Discussion

Mechanistically, LST1 is unlikely to function as a classical efferocytic receptor; instead, it may modulate efferocytosis indirectly by influencing cytoskeletal remodeling, membrane tethering, and MHC-I–linked antigen handling in myeloid cells, thereby reshaping how apoptotic cargo is processed and presented. Although LST1 was elevated in granulocytes from T2DM samples and macrophages from ccRCC tissues, our data suggest that its expression is further increased in comorbid states and is tightly coupled to coordinated activation of tumor-associated antigen-presentation and immune-exhaustion signatures. Clinically, this implies that while LST1 reflects chronic inflammatory activation, it achieves greater diagnostic discrimination when combined with tumor-specific markers such as CA9, PBRM1, and HLA-exhaustion signatures.

Taken together, these findings support the view that LST1 upregulation is initially a secondary response to chronic metabolic and inflammatory stress; however, once elevated, LST1 amplifies efferocytic and MHC-I–mediated communication circuits in granulocytes and macrophages, thereby contributing to the development of an immunologically stressed and ultimately immunosuppressive microenvironment. At the pathway level, our observations are consistent with known pro-tumorigenic inflammatory mechanisms shared between metabolic disease and carcinogenesis, including IL-6, MIF, GALECTIN, and CXCL signaling. Thus, the LST1-centered efferocytosis–communication axis provides a plausible mechanistic bridge linking long-term metabolic inflammation to immune remodeling in ccRCC.

The interplay between metabolic diseases and tumors is an expanding area of precision-medicine research, yet the immune-regulatory mechanisms underlying this comorbid relationship remain incompletely understood (29). Efferocytosis, a central process for clearing apoptotic cells (30), offers a biologically coherent point of convergence between these two diseases. By integrating single-cell transcriptomics from T2DM and ccRCC with machine-learning analyses, we propose that LST1-mediated efferocytosis and immune communication represent a core mechanism underlying comorbidity. This hypothesis is strengthened by our experimental findings: LST1 knockdown reduced ccRCC cell migration and colony formation (**Figures 7A–E**) and inhibited tumor growth *in vivo* (**Figures 7H–J**). This analytical framework—linking cellular behaviors, gene expression features, and signaling pathways (31–33)—not only clarifies LST1’s function within each disease but also outlines an immune-continuity pathway across metabolic dysfunction and malignancy (34).

The shift in dominant efferocytic cell type from granulocytes in T2DM to macrophages in ccRCC (35) has not been systematically described previously. Despite differences in participating cell types, the underlying regulatory circuits of efferocytosis appear highly conserved across diseases (36, 37). LST1 exemplifies this shared regulatory machinery. As a leukocyte-specific gene encoded in the MHC-III region, LST1 has been linked to T-cell activation, inflammatory responses, and adhesion (38). Our results identify a previously unrecognized role of LST1 as a regulator within the efferocytosis–communication axis, and this regulatory effect is conserved across both disease states (**Figures 6C, F**).

Using the SuperPC model, we identified LST1 among 58 candidate genes and further validated its specific expression in granulocytes and macrophages at the single-cell level (39). High LST1 expression correlated with increased activation of the MHC-I pathway in both T2DM and ccRCC (40, 41). Additionally, enriched ligand-receptor interactions in the granulocyte–T-cell and macrophage–NK/T-cell axes (42, 43) suggest that elevated LST1 expression intensifies immune signaling, promoting antigen presentation, adhesion, and chemokine-driven communication.

Beyond these observations, the LST1-associated signaling network encompasses multiple immune-regulatory functions, including cytokine modulation (e.g., IL-6, TNFSF13B) (49), antigen presentation (e.g., HLA-E–CD94) (50), cell adhesion (e.g., PECAM1–PECAM1) (51), and immune regulation (e.g., LGALS9–CD45) (52). These coordinated features indicate that LST1 acts not only in efferocytic recognition but also as a driver of broader immune-evolutionary trajectories relevant to comorbidity.

The consistency of LST1-mediated communication in both T2DM and ccRCC—particularly through MHC-I, TGF-β, CXCL, and ICAM1 pathways (44–46)—supports a unified immunological bridge between metabolic dysregulation and tumor progression. From this perspective, chronic LST1-driven activation of granulocytes in T2DM may promote sustained antigen presentation and inflammatory priming, which is subsequently mirrored and intensified by macrophages in ccRCC tissues, ultimately contributing to immune escape and immunosuppression (47).

These findings have two major clinical implications. First, diagnostic value: LST1 shows high accuracy in identifying T2DM-ccRCC comorbidity (AUC > 0.745), enabling early recognition of tumor risk in diabetic patients. Second, therapeutic potential: targeting LST1 or its downstream MHC-I pathway may both alleviate chronic inflammation in T2DM and reverse immune suppression in ccRCC, providing a unified therapeutic strategy.

Prior studies have attributed increased cancer risk in T2DM to metabolic burden or hyperinsulinemia; however, our results highlight immune-communication alterations during low-grade inflammation as an additional mechanistic route to carcinogenesis (48). The LST1-centered network identified in this study integrates cytokine signaling, antigen presentation, adhesion, and immune regulation, reinforcing its role as a key regulatory hub in T2DM–ccRCC comorbidity.

This study further benefits from a comprehensive research strategy that integrates single-cell multi-omics, immune-function annotation, immune-subtype mapping, and communication-network reconstruction (53). The strong cross-cohort consistency of the SuperPC model (54) supports the robustness of LST1 as both a predictive biomarker and a mechanistic contributor. Nevertheless, limitations remain. Although our data demonstrate that LST1 regulates the MHC-I axis and efferocytic communication (**Figures 6C, F**; **7F, G**), direct molecular interactions between LST1 and efferocytosis-related receptors (e.g., MerTK, TIM4) have not yet been validated. Future Co-IP and ChIP assays are required to characterize these interactions. Additionally, the use of public transcriptomic datasets introduces constraints related to sample size, heterogeneity, and clinical annotation. Multi-center single-cell datasets will be valuable for validating the spatial-temporal dynamics of LST1. Whether LST1 functions similarly in other chronic metabolic disorders or cancers also warrants further investigation.

## Conclusion

In summary, our study identifies LST1 as a novel pivotal regulatory factor that links efferocytosis, immune signaling, and disease progression in the comorbidity of T2DM and ccRCC. We also propose a cross-disease immune regulation hypothesis centered on the MHC-I pathway. This investigation elucidates the immunological underpinnings of the convergence of metabolic disorders and cancer, offering both a conceptual framework and potential molecular targets for the prospective design of precise therapeutic interventions targeting comorbidity pathways.

## Data Availability

The original contributions presented in the study are included in the article/**Supplementary Material**. Further inquiries can be directed to the corresponding authors.
