# Supplementary material for: LST1: a novel biomarker for efferocytosis in the co-occurrence of type 2 diabetes mellitus and clear cell renal cell carcinoma
Source: Front Immunol. 2025 Dec 19;16:1737749. doi: 10.3389/fimmu.2025.1737749 (PMC12757293; doi:10.3389/fimmu.2025.1737749)
Supplement: Supplementary file 1 [file DataSheet1.docx]

LST1: A Novel Biomarker for Efferocytosis in the Co-Occurrence of Type 2 Diabetes Mellitus and Clear Cell Renal Cell Carcinoma

Yuru Yang ^1,#^, Tingting Chen ^1,#^, Zhicheng Xu ^1,^ ^2,#^, Jialing Cai ^1^, Wenli Wang ^1^, Lan Pan ^3^, Xiaotian Cheng ^2,*^, Andong Wang ^1,*^

^1^ School of Pharmacy, Nantong University, Nantong, Jiangsu 226001, People’s Republic of China;

^2^ Department of Pharmacy, Yancheng Clinical College of Xuzhou Medical University & First people’s Hospital of Yancheng, Yancheng, Jiangsu 224001, People’s Republic of China;

^3^ College of Traditional Chinese Medicine, Xinjiang Medical University, Urumqi 830017, People’s Republic of China

* Correspondence: Andong Wang: [wangandong19891220@163.com](mailto:lingbai@163.com); Xiaotian Cheng: [cxt13485218739@163.com](mailto:cxt13485218739@163.com);

^#^  These authors contributed equally to this work and shared first authorship

| **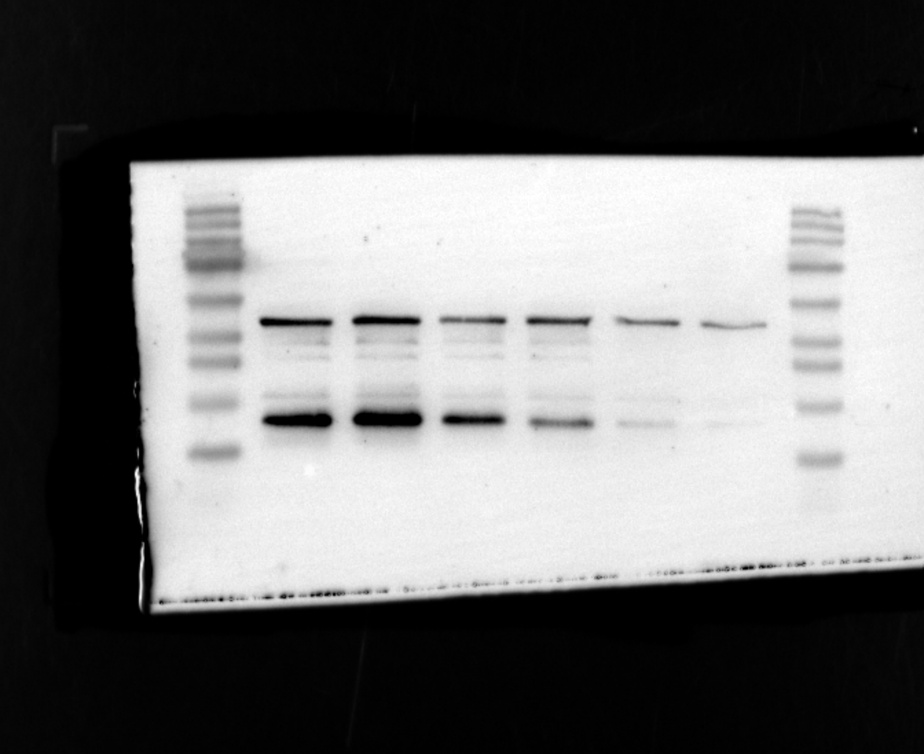** |
| --- |
| Figure S1 Western blot on LST1 |

| **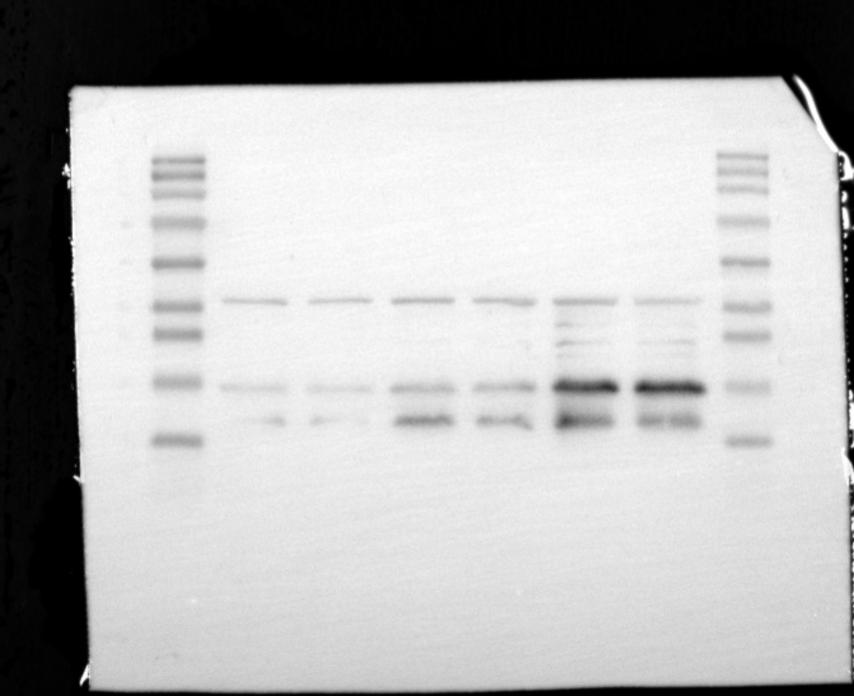** |
| --- |
| Figure S2 Western blot on BAX |

| **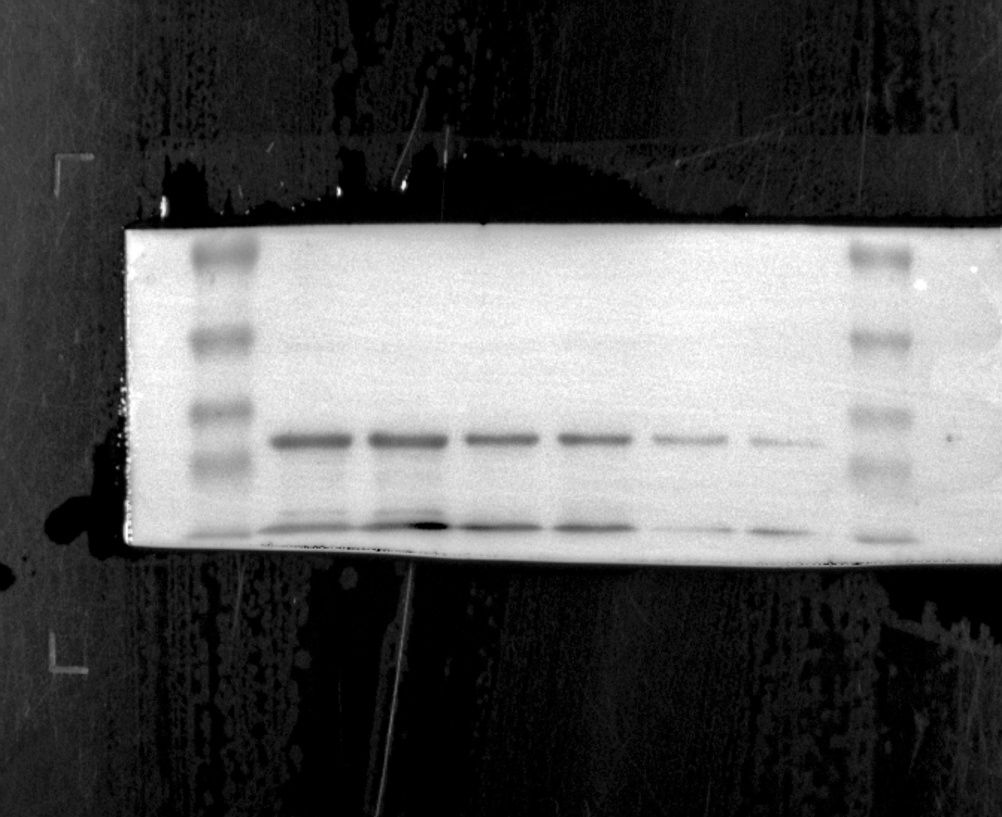** |
| --- |
| Figure S3 Western blot on BCL2 |

| **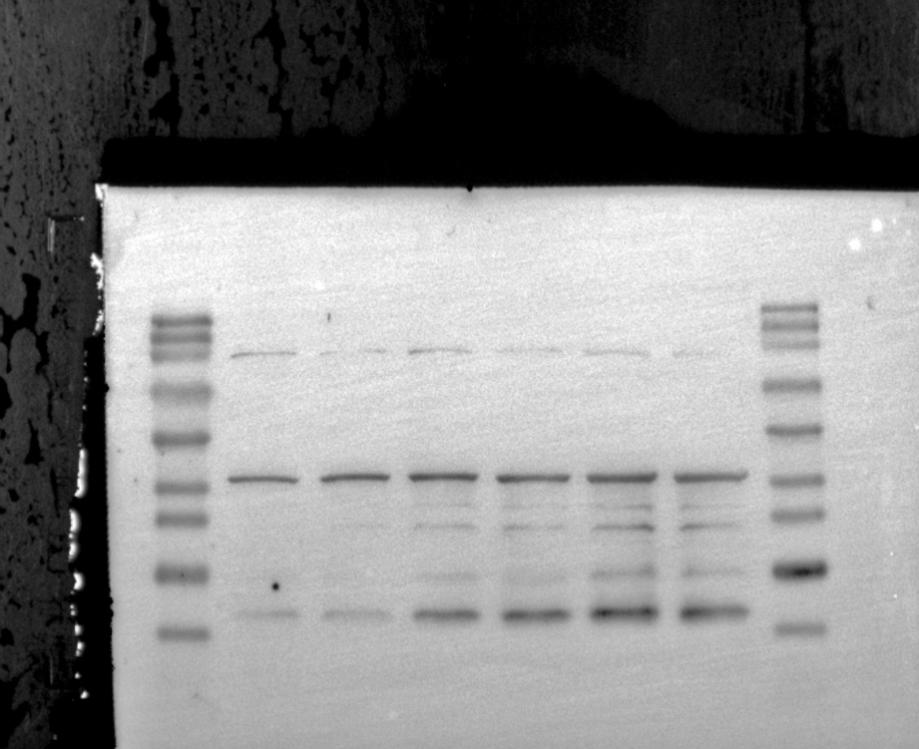** |
| --- |
| Figure S4 Western blot on Cleaved Caspase 3 |

| **** |
| --- |
| Figure S5 Western blot on MHC-1 |

| **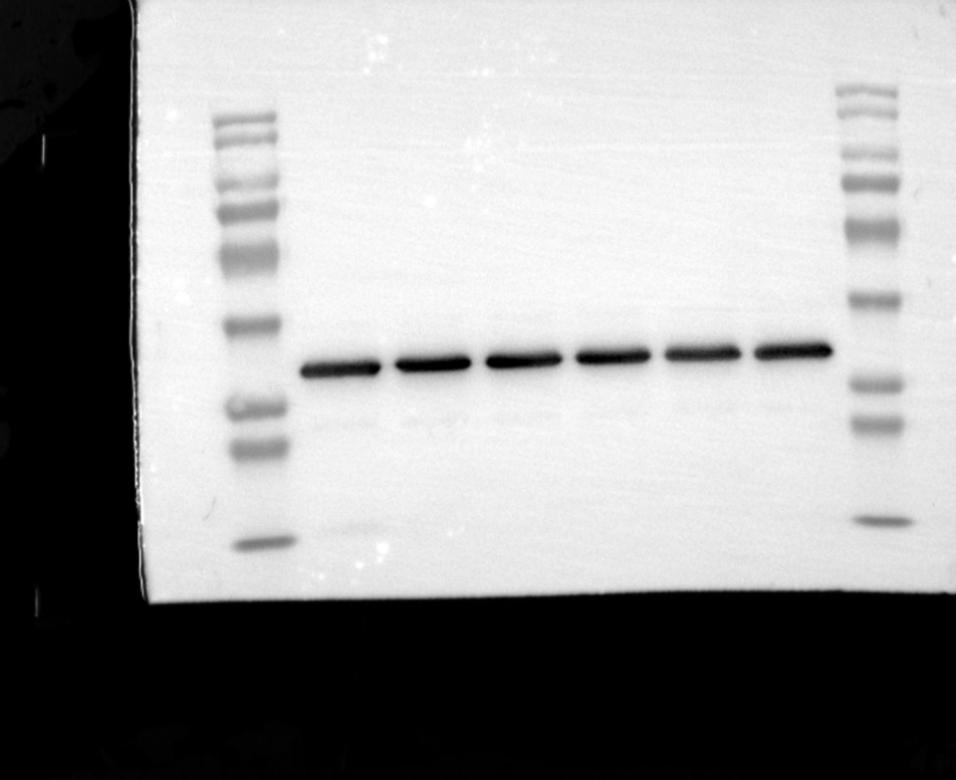** |
| --- |
| Figure S6 Western blot on GAPDH |
